# Report of the Diseases of Edinburgh for April, 1810

**Published:** 1810-06

**Authors:** John Roberton


### *Report of the Diseases of Edinburgh for April,* 1810.

By John Roberton, M. D.

Till about the beginning of the last third of the month, the weather was, for the most part, either wet or cold, and often both at once, with occasional hard gales of wind. The weather, somewhat similar to that of summer, burst upon us with unexampled fineness. But this only lasted two or three days, when the wind shifting into the east, thick fogs pervaded the whole city. For two or three of the last days of the month, however, the weather again became very pleasant.

The barometer was in general low till near the end of the month, when it rose considerably, and continued so till the end.

The thermometer was, unless one day or so from time to time, rather high.

The most prevailing diseases have been chin-cough, a general inflammatory state of the system, stomach complaints and diarrhœa, catarrh both acute and chronic, small-pox, croup, rheumatism, and cynanche tonsillaris.

Measles, which occupied some of our attention last month, seem to have entirely disappeared; at least, no cases of that disease have come under my observation for some time past. Most of the other diseases of last month still prevail, though somewhat various in severity. The chin-cough seems increased both in extent and severity. Indeed, several patients have died of that affection. It is in general only to be met with among infants and children, yet several cases of considerable violence have been met with in persons advanced in life.

The general inflammatory state of the system does not seem diminished in extent, but it now appears more commonly as affecting the whole system than any particular organ of it.

The bowel and stomach complaints, though, perhaps, less extensively prevalent, do not seem at all abated in their violence. Some cases have occurred to me of the most obstinate nature, defying, for a considerable length of time, every application that could be devised for their removal. Temporary relief was easily obtained, and the patients seemed, not unfrequently for several days, entirely free from every symptom of the disease. But, without any evident cause, the most severe griping, accompanied and followed by purging, recurred, which required the very utmost attention, both on the part of the patient and medical attendant, to accomplish a permanent cure.

Although the cases of acute catarrh are very general, yet none of them seem attended by any thing more than temporary inconvenience. A few doses of purgative medicine, with, in severe cases, confinement to the house for a few days, is generally followed by a complete cure. The chronic cases of this disease are, as formerly stated, much more troublesome, rather admitting of temporary alleviation than of complete removal.

The

The fevers, as usual, are still common, and in many instances, fatal.

The following diseases seem added to our list since last month.

Small-pox, various cases of which are of the very worst kind. Indeed, Edinburgh still furnishes this disease, though by no means so extensively as previous to the inoculation by the cow-pox, in every variety of severity from the mildest distinct pustule to the disease in its most confluent and dreadful form. From these circumstances, it is unnecessary to add, that of course various of these confluent cases have terminated fatally. So much for the incorrigible stubbornness of bigotted individuals. One child, affected in this way, which I had an opportunity of seeing once or twice before it terminated fatally, had been previously inoculated with the cow-pox. Owing, perhaps, to the inertness of the matter, the family surgeon had performed the operation four different times, but at neither of these, was any pustule produced. The child's grandmother, one of those old matrons who prove by their ignorance and impertinence, an indescribable annoyance to the medical attendant, would not allow the operation to be repeated, because forsooth it occurred to her (and what convinced her of the certainty of her opinion was, that she had dreamt about it) that the child was not *ordained* to be affected with the cow-pox. The other members of the family were prevailed on to listen to the remonstrances of this dreamer, and the consequence of this was, that the natural small-pox, of the very worst kind, affected the child, of which, as just stated, he died.

The croup, though by no means a very common disease of this place, has of late appeared much more generally than usual. Almost all the cases have been very severe, and many of them have terminated fatally. Most of those which did not, ran their course very rapidly, and even the most active practice could not avert such a termination. It is scarcely to be credited, in some of these cases, how immense a quantity of calomel was administered without visibly affecting the patient, even in the very slightest degree. In these cases, of course, no alleviation of the symptoms took place; but where salivation was induced, the patients, almost in every instance, very speedily recovered.

Rheumatism, both chronic and acute, has been of pretty frequent occurrence. The acute seems, in general, confined to the young and otherwise healthy part of the community, community, and especially to those who, from their occupation, are exposed to much personal exertion. The latter is more common among the old and debilitated. In the former, purging and sudorific medicines, with, after the violence of the symptoms has abated, the judicious application of friction, either with a brush alone, or with any of the stimulating balsamic applications, is of the greatest service. The latter does not require the previous drenching plan, but seems more successfully treated by the friction, even from the commencement of the symptoms. I some time ago gave a formula for pills, which, from the obstinacy of the symptoms, when internal medicines are necessary, I have found extremely efficacious. I need not now repeat it.

Various cases of cynanche tonsillaris have occurred in practice, and of various degrees of severity. The milder cases I have in previous reports had occasion to state, were easily removed by the repeated use of purgative medicines. But other cases have required not only these, but the strictest attention to confinement in the house, with the use of gargles, the application of leeches as near as possible to the parts affected, and a blister all over the thorax. Independently, however, of these active steps, several cases which have come under my observation have proceeded to suppuration.

*Princes Street.*

---

In answer to the private Correspondent, who signs himself A Friend to Improvements, and who says he is a reader of the Medical and Physical Journal, but who has not given his address, Dr. Roberton conceives the remarks in his letter, respecting the case of seminal emission, to be rather complicated, and of course of such a nature, that a personal interview with the gentleman can alone admit of their being properly and satisfactorily explained. Dr. Roberton, therefore, judges it most prudent to decline even attempting to answer them in the Medical and Physical Journal as requested; and he does this more readily, as, about the beginning of August, he will be in London for one or two days, when he will be happy personally to communicate to his correpondent whatever information on the subject he may stand in need of.

Dr. James Roberton, Leicester Street, Dr. Roberton's brother, will inform the gentleman, within a day or two of the time when he will be in London.

INTEL-

